# Quality Variation of Goji (Fruits of *Lycium* spp.) in China: A Comparative Morphological and Metabolomic Analysis

**DOI:** 10.3389/fphar.2018.00151

**Published:** 2018-02-26

**Authors:** Ruyu Yao, Michael Heinrich, Yuanfeng Zou, Eike Reich, Xiaolei Zhang, Yu Chen, Caroline S. Weckerle

**Affiliations:** ^1^Department of Systematic and Evolutionary Botany, University of Zurich, Zurich, Switzerland; ^2^Research Cluster Biodiversity and Medicines, Centre for Pharmacognosy and Phytotherapy, UCL School of Pharmacy, University College London, London, United Kingdom; ^3^Natural Medicine Research Center, College of Veterinary Medicine, Sichuan Agricultural University, Chengdu, China; ^4^CAMAG Laboratory, Muttenz, Switzerland; ^5^National Heart and Lung Institute, Faculty of Medicine, Imperial College London, London, United Kingdom; ^6^Agronomy College, Sichuan Agricultural University, Chengdu, China

**Keywords:** *Lycium*, goji, metabolomics, HPTLC, ^1^H NMR, climatic region, chemometric

## Abstract

Goji (fruits of *Lycium barbarum* L. and *L. chinense* Mill.) has been used in China as food and medicine for millennia, and globally has been consumed increasingly as a healthy food. Ningxia, with a semi-arid climate, always had the reputation of producing best goji quality (*daodi* area). Recently, the increasing market demand pushed the cultivation into new regions with different climates. We therefore ask: How does goji quality differ among production areas of various climatic regions? Historical records are used to trace the spread of goji production in China over time. Quality measurements of 51 samples were correlated with the four main production areas in China: monsoon (Hebei), semi-arid (Ningxia, Gansu, and Inner Mongolia), plateau (Qinghai) and arid regions (Xinjiang). We include morphological characteristics, sugar and polysaccharide content, antioxidant activity, and metabolomic profiling to compare goji among climatic regions. Goji cultivation probably began in the East (Hebei) of China around 100 CE and later shifted westward to the semi-arid regions. Goji from monsoon, plateau and arid regions differ according to its fruit morphology, whereas semi-arid goji cannot be separated from the other regions. *L. chinense* fruits, which are exclusively cultivated in Hebei (monsoon), are significantly lighter, smaller and brighter in color, while the heaviest and largest fruits (*L. barbarum*) stem from the plateau. The metabolomic profiling separates the two species but not the regions of cultivation. *Lycium chinense* and samples from the semi-arid regions have significantly (*p* < 0.01) lower sugar contents and *L. chinense* shows the highest antioxidant activity. Our results do not justify superiority of a specific production area over other areas. Instead it will be essential to distinguish goji from different regions based on the specific morphological and chemical traits with the aim to understand what its intended uses are.

## Introduction

In Chinese medicine, “*daodi* medicinal material” (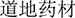, *dào dì yào cái*) spells out the relationship between medicinal material and one specific region with its traditional culture, human behaviors, and environmental conditions. Generally speaking, a *daodi* herbal medicine is derived from specific germplasm, a specific geographic location, and cultivated and processed with particular technologies with a long history. It is commonly recognized to be of high, stable quality, and reliable efficacy (based on traditional medicinal concepts), and has a longstanding, good reputation (Guo, 2004; Xiao et al., 2009; Zhao et al., 2012). However, nowadays the *daodi* concept is often not accepted as sufficient for defining good quality and as evidence for a species’ therapeutic benefits. The prevalence of herbal medicines for health purposes, such as food supplements and neutraceuticals, stimulates the botanical products market (Dillard and German, 2000; Lachance and Das, 2007; Knöss and Chinou, 2012; Hilton, 2016). For the widely consumed herbal medicines, *daodi* areas cannot produce enough plant material to meet the market demands; therefore, new cultivation areas are developed. Therefore, studies on the authentication of a species in the context of the production areas, quality criteria, as well as standardized plantation and processing techniques are increasingly important (Xiao et al., 2009). However, the constitutions of herbal medicines are influenced by a wide range of factors, such as meteorological conditions, environment and processing method (Donno et al., 2012, 2016b; Zhang et al., 2012; Qi et al., 2016; Shen et al., 2016). As a result, the authentication and quality control of botanical products become even more difficult.

This is the case for goji (*Lycii frutus*, fruits of *Lycium barbarum* L. and *L. chinense* Mill.), which has been used as health food and traditional medicine for millennia in China and other regions of the world (Yao et al., 2018a). Recently, numerous phytochemical and pharmacological studies focus on its health benefits, and support its use as functional food (often sold under the marketing concept of an alleged “superfood”) (Yao et al., 2011; Chang and So, 2015; Qian et al., 2017).

**FIGURE 1:**
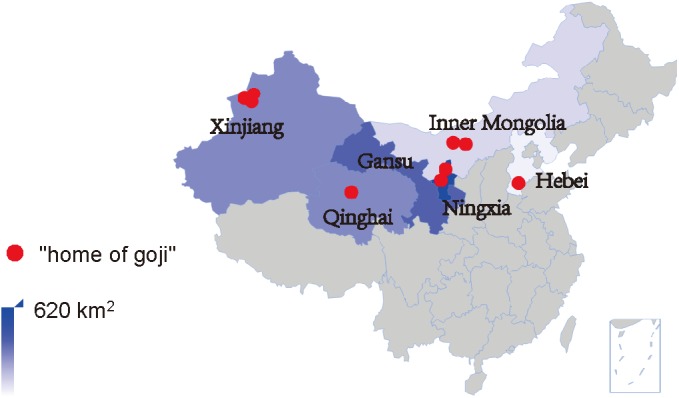
Goji cultivation areas and “home of goji” in China. Up to 2015, the total cultivation area was estimated to be 1500 km^2^, and nine cultivation areas were labeled as “home of goji.”

While Ningxia is recognized as the *daodi* region of goji, increasing market demands pushed the cultivation into new regions in China and goji fields now stretch over different geographical and climatic environments between 82°E and 115°E, 30°N and 45°N (Xu et al., 2014; Cao and Wu, 2015). These include temperate monsoon climate (Hebei), temperate continental semi-arid climate (Ningxia, Gansu, and Inner Mongolia), plateau continental climate (Qinghai), and continental arid climate (Xinjiang). These different environmental conditions influence both the appearance and the metabolite profile of goji (e.g., amount of polysaccharides, flavonoids, betaine, and carotenoids) (Zhang et al., 2012; Lin, 2013; Liu X.X. et al., 2015; Shen et al., 2016). Furthermore, different species and cultivars are cultivated in different areas: *L. barbarum* cultivars (e.g., *Ningqi* series) are widely cultivated while *L. chinense* is only cultivated in Hebei (Cao and Wu, 2015). In recent years, many of the new cultivation areas with different climates have been officially labeled as “home of goji” (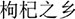, *gǒu qǐ zhī xiāng*) by different governmental bodies, a category distinct from *daodi* (**Figure 1**). This label stands for standardized production including growing it on large-scale fields and for high quality products.

Thus, both “*daodi”* and “home of goji” stand for good quality connected with a specific region. As these regions have different climates, one must ask*: How does goji differ among production areas of distinct climatic regions?*

Traditionally, good goji quality was defined as: Large berries with red color, mild texture, few seeds, and sweet taste (Chinese Pharmacopoeia Commission, 1963). Such morphological characteristics are largely used by goji traders for sensory identification. Recently, goji quality assessment also has relied on a few marker compounds, such as polysaccharides and betaine (Korea Food and Drug Administration, 2014; Chinese Pharmacopoeia Commission, 2015a), and HPTLC flavonoid fingerprint for species identification (European Directorate for the Quality of Medicines and Healthcare, 2017). Since berries from different species and cultivars are anatomically and morphologically similar, molecular identification, infrared spectroscopy, chemical fingerprint, and bioactivity are also used for identification or evaluation (Zhang et al., 2001, 2016; Yao et al., 2010; Xin et al., 2013; Donno et al., 2015).

In our study, we also include metabolomic profiling for quality assessment, which has not been used for goji yet. Chemometric approaches with chromatographic fingerprinting are known to be effective metabolomic methods for quality control of herbal medicine (Donno et al., 2016a; Guo et al., 2017; Liu et al., 2017; Martinez-Frances et al., 2017). Bioactivity-based characteristics are good quality indicators too, as they are pharmacologically relevant (Walch et al., 2011; Liu et al., 2017). Since goji was reported to have health protective effects against oxidative stress, and was recommended as a natural antioxidant (Donno et al., 2015; Benchennouf et al., 2017), we assume that antioxidant effects are an adequate quality marker.

Our research questions / objectives and the resulting research strategy are:

Does goji from climatically different production areas show different quality characteristics? Can we correlate quality measurements with specific production areas? We focus on the following quality measurements, which are then correlated with the four main production areas: fruit morphology, sugar and polysaccharide contents, antioxidant activity, and metabolomic profiling.
At least two species are traded widely as goji, requiring an understanding of the species similarities and differences. For this comparative characterisation and identification we use HPTLC.
To understand the spread of goji production in China over time we use historical records.

## Materials and Methods

### Historical Literature Study

Information about historic goji cultivation areas was retrieved from the 32 ancient Chinese herbals used in our previous study (Yao et al., 2018a); and the toponymes were cross-linked to contemporary maps. For developments in recent decades, the search engine Baidu^1^ was employed, using “
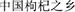
” (“home of Chinese goji,” *zhōng guó gǒu qǐ zhī xiāng*) as keyword; Then, the source information was tracked down. All data were integrated and maps were generated with R 3.4.1 and the R package REmap (Liang, 2015; R Core Team, 2017).

### Plant Material

Fieldwork was carried out by the first author for several weeks between August 2014 and September 2016 and goji samples were collected in the main production areas in China: Ningxia, Qinghai, Xinjiang, and Hebei. In total 51 fruit samples of *L. barbarum* and *L. chinense* were collected directly from the farmers, or were offered by institutions (Ningxia Qixiang Biological Foodstuff Co., Ltd., Hebei Julu Shengying Gouqi Co., Ltd., Xinjiang Gouqi Development and Management Center of Jinghe County, Jinghe Guokang Gouqi Specialized Farmers Cooperatives), or were bought in the Zhongning Goji Distribution Center, Julu Goji Yinhua Market, Xinjiang Jinghe Goji Market, Chengdu Hehuachi Chinese Herbal Medicine Market, and An’guo Chinese Herbal Medicine Market. This includes *L. chinense*: ten samples from the monsoon region; and *L. barbarum*: 25 from the semi-arid region, nine from the plateau region, and seven from the arid region. An authenticated reference standard sample of *L. barbarum* fruits was bought from the National Institute of Food and Drug Control of China, batch No. 121072-201410. Fruits with voucher specimens were collected in Zhongning County of Ningxia, Julu County of Hebei, and the National Gouqi Germplasm Garden in Yinchuan City of Ningxia. Totally, 24 specimens are deposited in the herbarium of the University of Zürich and ETH Zürich (Z+ZT).

### Morphological Identification

Identification of the specimens is based on the Flora of China (Wu et al., 1994) and *Lycium* specimens from the China National Herbarium (PE): The barcode No. of the referred specimens are 01882829, 00633675, 00633726, 00031413, 00031311, 00031382, 01432314, 00031381. Fruits of the following two specimens were used as reference for HPTLC fingerprint analysis: Z.000106520 (*L. chinense* Miller) and Z. 000106530 (*L. barbarum* L.).

### Chemicals and Reagents

Methanol (HPLC Ultra) was purchased from Roth; Ethyl acetate (98%+), Formic acid (99.5%), Glacial acetic acid (99.5%) were bought from Acros Organics; Dichloromethane (DCM) (99.9%+), Dimethylsulfoxide-d6 (DMSO-d_6_), 4,4-dimethyl-4-silapentane-1-sulfonic acid (DSS), and Trifluoroacetic acid (TFA) (99%) were obtained from Sigma; all reagents and chemicals are of analytical grade. NP reagent: 1 g Diphenylborinic acid aminoethylester in 200 ml Ethyl acetate. PEG reagent: 10 g Polyethyleneglycol-400 in 200 ml Dichloromethane.

### Morphological Measurements

Fruits were placed in a drying oven at 60°C for 6 h then were cooled down overnight. High-resolution images of the fruits were obtained by using an image scanner (Epson Perfection V750 Pro, United States). Length and width of each fruit were measured in Adobe Photoshop by using the ruler tool, which was done by selecting a smallest rectangle to cover the fruit outline and recording the length and width of the corresponding rectangle. Color parameters of the fruit images were represented by RGB values which were measured as follows: A smooth and clean area on the fruit image was selected such that its color was representative of the whole fruit; then, the RGB value of this area was read from the information toolbox using the color sampler tool in Photoshop.

For weight measurement 50 fruits per sample were randomly selected and weighed. This was repeated three times per sample. If the difference between any two of the three measurements was over 5.0%, another batch of 50 fruits was weighed. The process was repeated until there were three measurements with differences below 5.0%.

Data were analyzed with R 3.4.1 in RStudio 1.0.153. R packages of ggplot2, multcomp, and MASS were used (Venables and Ripley, 2002; Hothorn et al., 2008; Wickham, 2009; R Core Team, 2017).

### HPTLC Fingerprint Analysis

HPTLC fingerprint analysis was applied on all samples based on the monograph of *Lycii fructus* (04/2015:2612) in the European Pharmacopoeia 9.0 (European Directorate for the Quality of Medicines and Healthcare, 2017), with slight modification.

Test solution was prepared as follows: To 0.10 g of powdered goji, 7 ml of water were added and sonicated for 10 min at room temperature. After centrifugation (5000 r/min for 5 min), 4 ml of the supernatant was loaded onto a 6 ml solid phase extract (SPE) C18 cartridge (Strata C18-E, Phenomenex, United States) that had been pre-treated first with 3 ml of methanol, dried, and then with 3 ml of water (not dried). The loaded and dried cartridge was twice washed with 1 ml of water-methanol (90:10) and dried. The test solution was obtained by elution of the cartridge with 1 ml of methanol. During loading, clean-up and elution the flow rate of the solvent should not exceed 120 drops per minute.

A CAMAG HPTLC system (Muttenz, Switzerland) was employed, with an automatic TLC sampler 4 (AST 4) for application, an automatic developing chamber 2 (ADC 2) for developing, a chromatogram immersion device III for derivatization, a TLC plate heater III, a TLC visualizer 2 for imaging and a software visionCATS 2.0 for data analysis. Strata C18-E (55 μm, 70 A, 500 mg/6 ml) solid phase extraction cartridges from phenomenex were used for sample preparation, and the plates used were HPTLC glass 20 cm × 10 cm, Si 60 F_254_ (Merck, United States).

Application parameters were: Spray gas = air; Sample solvent type = methanol; filling speed = 15 μl/s; predosage volume = 200 nl; retraction volume = 200 nl; rinsing cycles/vacuum = 1/4 s; filling cycles/vacuum = 1/0 s; rinsing solvent name = methanol; nozzle temperature = unheated; rack in use = standard. And 10 μL of test solution and 2 μl of reference solution were applied as bands of 8.0 mm, while the application position was 8.0 mm from the lower edge of the plate; first position X: 20.0 mm, distance: 11.4 mm.

Developing conditions were: Humidity control: 33% with MgCl_2_; saturation: 20 min with filter paper; developing distance from application position/lower edge: 62/70 mm; developing solvent: Ethyl acetate, water, acetic acid, formic acid 100:27:11:11; developing time: 20 min; plate drying: 5 min.

Derivatization was done with chromatogram immersion device III at speed of 5 with time of 0 s. Derivatization reagents: NP regent and PEG regent. The developed plate was preheated at 100°C for 3 min; the warm plate was treated with NP reagent, and photographed when dry; treatment with PEG reagent followed, and pictures were taken after 5 min. Images were taken at conditions of “auto capture, Auto, level 85%, Band.” The images were analyzed with visionCATS 2.0.

### ^1^H NMR Profiling

For the metabolomic profiling of all samples, the method of Booker et al. (2016) was modified: 0.0500 g of goji (fine powder, freeze dried) in a 1.5 ml centrifuge tube with 0.900 ml DMSO-d_6_ and 0.100 ml of 0.100 g/ml DSS solution. After vortexing (Rodamixer, United Kingdom) for 30 s, the samples were sonicated in an ultrasound bath (XB22, Fisher, United Kingdom) for 20 min. Then, the solution was centrifuged for 5 min at 14,000 rpm (EBA 21, Hettich, Germany). Supernatant (0.600 ml) was transferred into a 5 mm diameter NMR spectroscopy tube for ^1^H NMR analysis (AV 500, Bruker BioSpin GmbH, Germany). The parameters were: solvent = DMSO, temperature = 300, experiment = 1D, number of scans = 128, pulse width = 13.9, acquisition time = 3.172, spectrometer frequency = 500.13, nucleus = ^1^H.

Since the samples could not be analyzed in one group, they were separated into five batches. To ensure the stability of the measurement, in every batch we took one sample for triplicated extraction and testing; one of the samples was analyzed in each batch.

The data were processed by MestReNova 9.0 (Mestrelab Research S.L., Spain). After phase correction and baseline correction, we calibrated the chemical shift reference by adjusting the first right DSS peak at 0.000 ppm. The spectrum was normalized by setting the total area of peaks (δ = 10.00–0.000 ppm) to 100.000. The binning function was applied to integrate the peak area by small buckets (0.040 ppm) with the method of “sum.” The data were exported as a “.csv” file and analyzed with R and R package ggplot2 (Wickham, 2009; R Core Team, 2017).

### Contents of Hydrolyzed Sugars and Polysaccharides

Moisture of all samples was measured according to the method No. 0831 of the Chinese Pharmacopoeia (Chinese Pharmacopoeia Commission, 2015b). 20 g of fruits were placed in an open aluminum box (with cover) into a drying oven at 105°C. After 4 h, the box was weighed every half hour until the weight no longer decreased, then all the dried samples were weighed and the moisture content calculated.

Hydrolyzed sugars were extracted according to the monograph of Xanthan Gum (04/2009: 1277) of the European Pharmacopoeia 9.0 (European Directorate for the Quality of Medicines, and Healthcare, 2017). To 0.050 g of goji powder in a thick-walled centrifuge tube, 2.0 ml of a 230.0 g/L solution of TFA was added, and shacked vigorously. The test tube was closed and heated at 120°C for 1 h. The hydrolysate was centrifuged, and 1.0 ml of the clear supernatant liquid transferred into a 10.0 ml flask, and 5.0 ml of water was added to obtain the test solution. Polysaccharides were extracted according to the monograph of *Lycii fructus* in the Chinese Pharmacopoeia (Chinese Pharmacopoeia Commission, 2010; Chinese Pharmacopoeia Commission, 2015a).

The contents of hydrolyzed sugars and polysaccharides were measured with the phenol-sulphuric acid method (Zou et al., 2011): glucose was used to plot a standard curve, and the final result was corrected with the moisture of the samples.

### Antioxidant Activity

Fifteen samples (three from monsoon, eight from semi-arid, two from plateau, and two from arid) were randomly selected, and each of them was prepared as follows: Fifty grams of fruits were extracted with 1000 ml of 95% ethanol, followed by 50% ethanol, and hot water. The supernatants were collected separately and concentrated with a rotary evaporator before frozen and dried in a freeze dryer (Zou et al., 2014). The extracts were named as A-95% EtOH, B-50% EtOH, and C-H_2_O, separately. Another 50.0 g of goji was extracted with 1000 ml of boiling water for the aqueous extract (Zou et al., 2014), and processed as above, and the extract was named as D-H_2_O.

The antioxidant activities of the extracts were tested using a modified ABTS method (Li et al., 2012). The obtained extracts were solved in 95% EtOH in the ratio: 1000.0, 800.0, 600.0, 400.0, and 200.0 μg/ml. Quercetin was used as positive control. IC_50_ values, the concentration of 50% radical-scavenging effect, were calculated by linear regression. Data were analyzed with R and package multcomp (Hothorn et al., 2008; R Core Team, 2017).

## Results and Discussion

### Ancient and Current Goji Distribution in China

**FIGURE 2:**
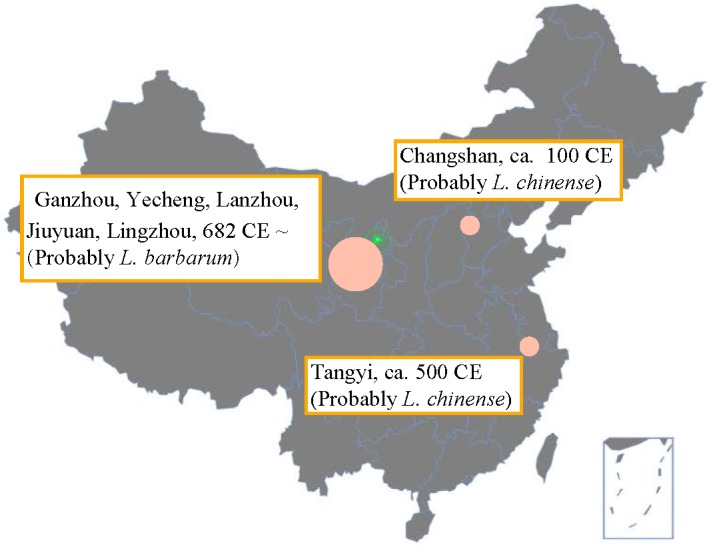
Goji cultivation map according to Chinese historical herbals (100 CE - 700 CE). The map was produced based on the historical maps of the Han and Tang Dynasty. Data are from *Shennong*’s herbal (ca. 100 CE), Mingyi Bielu (ca. 500 CE), and Qianjin Yifang (682 CE). Later, good quality goji was consistently ascribed to the semi-arid region.

The ancient distribution of goji cultivation areas is shown in **Figure 2**. The earliest goji record was found in the *Shennong*’s Herbal Classic describing that around 100 CE it grew in Changshan (now Hebei) during the Han Dynasty (Shang, 2008). According to the ascribed bitter taste we assume that they were describing *L. chinense*. Around 500 CE, goji was recorded to appear widely around Tangyi at south of Hebei (Su, 1981; Tao, 1986). Sun Simiao recorded 682 CE that goji grew in the semi-arid climatic region in Ganzhou, Yecheng, Lanzhou, Jiuyuan, and Lingzhou; he also described its cultivation technology and the sweet taste and superior quality of the berries compared to other regions (Sun, 1997). Very probably he referred to *L. barbarum*. From the Song Dynasty (10th-13th century) onward, goji was mentioned widely, and most records agreed that goji (probably *L. barbarum*) from semi-arid regions had the best quality (Wu, 1959; Tang, 1982; Li, 2003). Overall, goji cultivation seems to have begun in the East (Changshan) and later shifted westward to the semi-arid regions.

**FIGURE 3:**
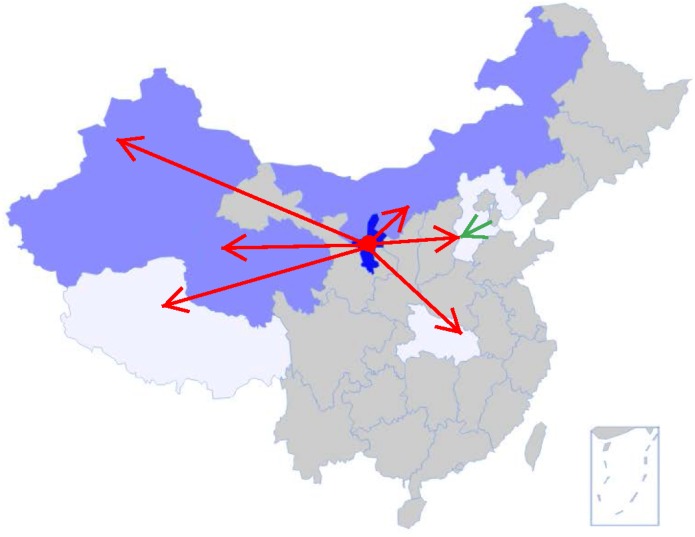
Development of goji cultivation since 1960s. Cultivars of *L. barbarum* were introduced in large scale from Ningxia (dark blue) to Inner Mongolia, Xinjiang, Qinghai (bright blue); and in lesser degree into Tibet, Hubei, and Hebei (white) (red arrows). The green arrow shows the expansion route of *L. chinense* westward within Hebei.

Since the 1960s, goji was introduced further west and north to the Inner Mongolia (semi-arid region), Qinghai (plateau region), and Xinjiang (arid region); in Hebei, cultivation shifted westward from Jinghai to Julu and Qinglong County (**Figure 3**), while *L. barbarum* was also introduced for cultivation (Cao and Wu, 2015). Since the Chinese pharmacopoeia exclusively accepts *L. barbarum* as medicinal goji (Chinese Pharmacopoeia Commission, 2015a), *L. chinense* is only locally cultivated. The fruits of *L. chinense* are sparsely consumed in China, but in Japan and Korea, the two species are used interchangeably (Korea Food and Drug Administration, 2014; Japanese Pharmacopoeia Editorial Committee, 2016).

In 1961, Zhongning County of Ningxia was assigned to be the unique national plantation for goji by the State Council of the P. R. China (Su, 2002). At a small scale, goji was also introduced to Hubei and Tibet in the 1970s and 2000s, respectively (Cao and Wu, 2015).

In recent years, the increase of goji consumption stimulated a drastic increase of goji cultivation and the total cultivation area in China now exceeds 1500 km^2^. As shown in **Table 1**, up to 2017, eight “home of Chinese goji” labels and one “home of Hebei goji” were attributed to cultivation areas in different climatic regions. The latter is probably due to the long cultivation history of the area and not to the size of production area which covers only around 4 km^2^. Both titles indicate the reputation of high quality products.

**Table 1:** An overview of “home of goji” in China until 2017^∗^.

Climatic region	Province	Cultivation location	Issued tittle	Issue department	Issue time
Monsoon	Hebei	Julu	Home of Hebei goji	HBG	2002
Semi-arid	Ningxia	Ningxia	Home of Chinese goji	SC	1995
		Zhongning		EFA	2000
	Inner	Hangjinhou Qi		SFA	2004
	Mongolia	Wulateqian Qi		EFA	2013
Plateau	Qinghai	Nuomuhong Farm		EFA	2015
Arid	Xinjiang	Jinghe		MA	1998
		Nongwu Shi		SFA	2001
		Jinghe		SFA	2004

^∗^*Semi-arid, temperate continental semi-arid climate; Arid, continental arid climate; Plateau, plateau continental climate; Monsoon, temperate monsoon climate. SC, the State Council of the P. R. China; MA, Ministry of Agriculture of P.R. China; SFA, State Forestry Administration of P.R. China; EFA, Economic Forest Association of China; HBG, Government of Hebei Province. (Cao and Wu, 2015; State Forestry Administration of P.R. China, 2017)*.

### HPTLC Fingerprint for Species Identification

**FIGURE 4:**
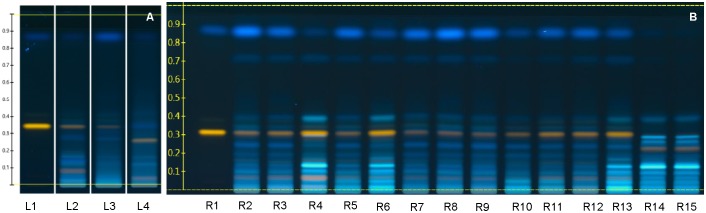
Goji flavonoid fingerprint by HPTLC. **(A)** References (L1, Rutin Rf = 0.34 and Scopletin Rf = 0.88; L2, *Lycii fructus* standard reference; L3, voucher specimen of *L. barbarum*; L4, voucher specimen of *L. chinense*); **(B)** Samples (R1, Rutin and Scopletin; R2 – R13, samples of *L. barbarum*; R14 – R15, samples of *L. chinense*). Derivatization was done with NP regent and PEG regent, and images were taken at 366 nm.

Fruits of *L. barbarum* and *L. chinense* cannot be distinguished readily (Xin et al., 2013). We therefore checked and confirmed the identity of all samples with HPTLC fingerprinting (**Figure 4**).

Flavonoids fingerprints of the same species are highly similar, indicating their chemical similarity, while fingerprints of the two species differ. The most obvious differences are: *L. barbarum* shows a yellow zone at Rf = 0.32 ∼ 0.35, which is rutin; while *L. chinense* lacks rutin, but has a yellow zone at Rf = 0.25 ∼ 0.27, which might be a derivative of rutin.

### Morphological Characteristics

**FIGURE 5:**
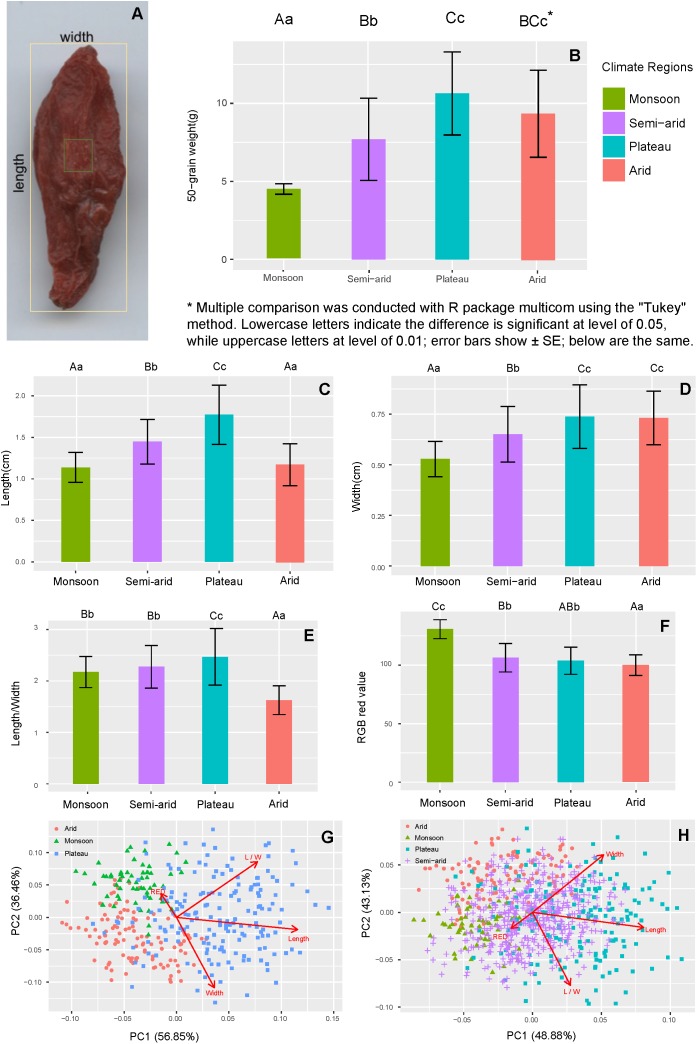
Comparison of morphological traits of goji from different climatic regions. **(A)** Example of measuring shape and color; **(B)** Average weight per 50 fruits; **(C)** Average fruit length; **(D)** Average fruit width; **(E)** Average length/width ratio of fruits; **(F)** Average redness of fruits; **(G)** Morphological PCA score plot without semi-arid region; **(H)** Morphological PCA score plot of all regions.

Length, width, length/width ratio, and redness are intuitive criteria used by stakeholders (cultivators, middlemen, and manufacturers) to evaluate goji quality. **Figure 5A** shows an image of goji. Samples from the semi-arid region cannot be separated morphologically from the other regions and were excluded for further analysis (**Figures 5G,H**). According to a quadratic discriminant analysis (QDA), the within training data classification error rate is 12.8%. Samples from monsoon, plateau and arid regions differ in their morphology (**Figure 5G**).

Overall, *L. chinense* fruit samples from Hebei are significantly lighter, smaller and brighter in color (**Figures 5B–D,F**), while the heaviest and largest fruits stem from the plateau (**Figures 5C,D**). In the plateau and arid region, the drastic temperature fluctuation between day and night seem to have a positive influence on fruit weight (Qi et al., 2016).

Commercial goji are normally categorized into six grades depending on the number of fruits per 50 g. The best grade contains 180–200 fruits per 50 g, and the lowest grade 980 fruits. **Figure 5B** shows a weight comparison of goji from different climatic regions.

The shape of goji fruits, which is measured by length/width ratio, differs significantly (*p* < 0.01) among regions (**Figure 5E**). Goji from the plateau region has the largest, while the arid region shows the smallest ratio. Goji from the plateau appears to be elongated oval or lanceolate, while samples from other regions are of shorter oval shape. This character is often used by stakeholders to identify the origin of goji. In the market, goji with larger berries tend to get higher prices. Therefore, farmers of the plateau region often press goji before selling it.

Redness is attractive for most consumers, unless it is too bright which may hint at sulfur treatment. In the monsoon region it is generally difficult to dry goji in the sun, and therefore sulfur fumigation is often applied to avoid degradation due to moisture. As a result, Hebei goji tends to be bright red in color. In the arid region sulfur is rarely used and goji redness is lower. The use of sulfur in semi-arid and plateau regions depends on the weather, whereas sulfur is applied in case of rain or lack of sun. In recent years, artificial drying rooms have been used increasingly to decrease the use of sulfur.

Fruit traits of goji are affected by temperature, humidity, duration of sunshine and altitude (Lin, 2013; Qi et al., 2016). However, fruit morphology may differ even within the same climatic region (Lei et al., 2013). Moreover, the drying process has an impact on the color (Ma et al., 2008). Goji from the semi-arid region, especially Ningxia, is recognized as having higher quality compared to other regions. However, from our morphological analysis goji berries from the semi-arid region are not distinguishable from berries from other regions and, therefore, it is possible to wrongly label goji from different regions as “Ningxia goji” to charge high prices.

### ^1^H NMR Based Metabolomic Profiling

**FIGURE 6:**
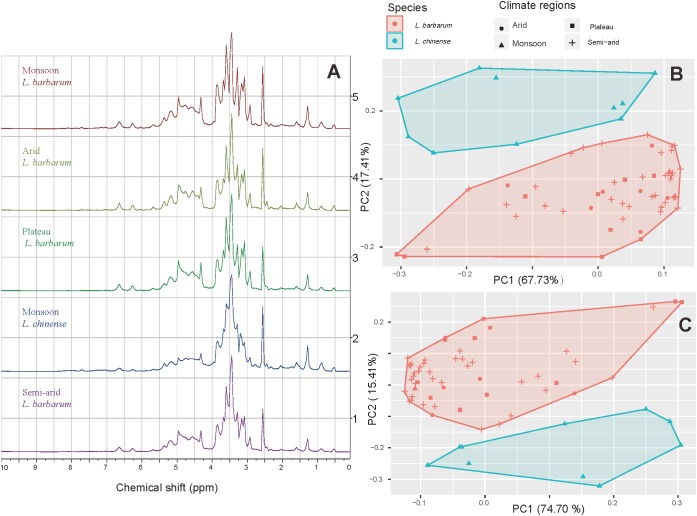
^1^H NMR spectra and PCA projections of the first two principle components. **(A)** Spectra of goji samples from different climatic regions (δ = 10.00 – 0.00); **(B)** Score plot based on δ = 10.00 – 0.00; **(C)** Score plot based on δ = 6.00 – 3.00.

With the metabolomic profiling we were able to separate the two species but not the samples from different cultivation regions (**Figure 6**). Differentiation of several other species according to their metabolomic profiles has been done before, e.g., *Rhodiola* spp. and *Curcuma* spp. can be separated successfully (Booker et al., 2014, 2016).

The typical spectra of goji from different climatic regions are shown in **Figure 6A**. Profile No.2 differs from the others in peak shape, especially between 2.50 and 5.00 ppm. PCA analysis with data from 0.00 to 10.00 ppm shows that the two species *L. barbarum* and *L. chinense* are separated by their metabolomics (**Figure 6B**). The region between 3.00 and 6.00 ppm, which importantly includes the signals from diverse sugars, turned out to differ strongly between the two species, which again becomes visible with PCA (**Figure 6C**); therefore, this part is a characteristic region for species identification.

Similar results were reported with a chemometric approach based on Fourier-transform infrared spectroscopy (FT-IR), by which goji from Hebei was discriminated from that of other regions, while others were clustered closely (Shen et al., 2016). HPLC was successfully used to differentiate between genotypes of hybrid cultivars between *L. barbarum* and *L. chinense*, as well as fruit samples of the same genotype but dried in different methods (Donno et al., 2016b). Furthermore, chemometric approach was applied to FT-IR, high performance size-exclusion chromatography (HPSEC), and pre-column derivatization high-performance liquid chromatography (PCD-HPLC), which indicated that polysaccharides of goji from different regions barely differed (Liu W. et al., 2015). These findings are supported by a recent research which shows that the molecular structure of polysaccharides from goji (*L. barbarum* only) of different regions is highly similar (Wu et al., 2015). This similarity is also shown by our metabolomic analysis, where *L. barbarum* samples from different cultivation areas cluster together, but differ from *L. chinense*.

### Hydrolyzed Sugar and Polysaccharides

**FIGURE 7:**
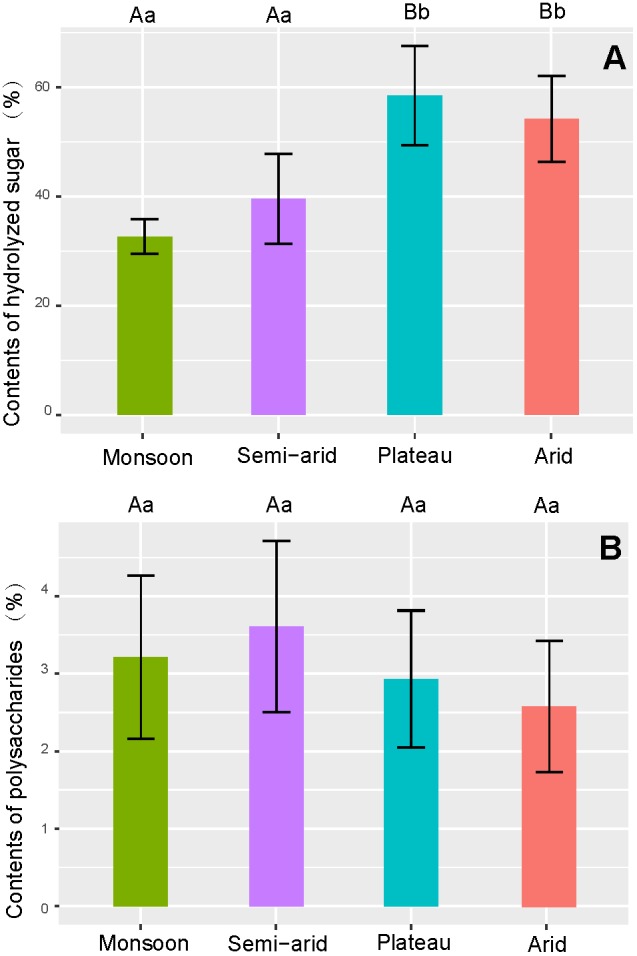
Comparison of sugar content in goji from different climatic regions. **(A)** Average contents of hydrolyzed sugar; **(B)** Average contents of polysaccharides.

*Lycium chinense* as well as samples from the semi-arid regions have significantly (*p* < 0.01) lower sugar contents compared to the plateau and arid region (**Figure 7A**). No differences in polysaccharide content could be found neither between the species nor the cultivation regions (**Figure 7B**).

Goji contains sugars at a relatively high level, and goji polysaccharides are recognized as main bioactive compounds (Xie et al., 2016; Yao et al., 2018b); as a result, sweetness and contents of polysaccharides are quality criteria of goji. The accumulation of sugar and polysaccharides in goji was reported to be affected by temperature, humidity, and altitude; variation even in the same climatic region is huge (Lei et al., 2013; Qi et al., 2016). Therefore, the differences among climatic regions are relatively small.

### Analysis of the Antioxidant Activity

While all the IC_50_ values are far higher than that of the positive control, we find differences among goji from different regions. *Lycium chinense* from the monsoon region shows the highest antioxidant activity for all preparations, while goji from the plateau shows for most preparations the lowest antioxidant activity, although differences are non-significant (*p* > 0.05) (**Table 2**). Thus, *L. chinense* seems to be more potent as a natural antioxidant.

**Table 2:** IC_50_ value (μg/ml) for scavenging ABTS⋅^+^ compared by regions^∗^.

Climate	A-95% EtOH	B-50% EtOH	C-H_2_O	D-H_2_O
Arid	90.95 ± 2.45 ab	66.00 ± 3.30 ab	76.60 ± 3.10 b	70.65 ± 6.40 ab
Monsoon	77.90 ± 3.27 a	37.50 ± 0.16 a	39.30 ± 0.49 a	44.10 ± 1.80 a
Plateau	108.30 ± 5.40 ab	72.50 ± 3.70 b	92.55 ± 3.55 b	71.85 ± 7.75 ab
Semi-arid	105.74 ± 14.91 b	56.41 ± 13.42 ab	84.59 ± 13.09 b	74.51 ± 14.73 b

^∗^*The IC_*50*_ value of quercetin (positive control) was 0.75 ± 0.01 μg/ml (mean ± SE). Multiple comparison was carried out with the method of “Tukey”; and in the same column, means sharing the same letters are not significantly different from each other (*p* > 0.05)*.

As traditional food supplement, goji has been used as medicinal liquor, i.e., soaked in spirit (40–70% EtOH), as condiment boiled with food, or as tea infused with boiling water. Our extracts imitate these traditional uses (**Table 3**).

**Table 3:** IC_50_ value (μg/ ml) for scavenging ABTS^⋅+^ compared by preparations^∗^.

Preparations	Monsoon	Semi-arid	Plateau	Arid
A-95% EtOH	77.90 ± 3.27 c	105.74 ± 14.91 c	108.30 ± 5.40 b	90.95 ± 2.45 b
B-50% EtOH	37.50 ± 0.16 a	56.41 ± 13.42 a	72.50 ± 3.70 a	66.00 ± 3.30 a
C-H_2_O	39.30 ± 0.49 ab	84.59 ± 13.09 b	92.55 ± 3.55 ab	76.60 ± 3.10 ab
D-H_2_O	44.10 ± 1.80 b	74.51 ± 14.73 ab	71.85 ± 7.75 a	70.65 ± 6.40 ab

^∗^*The IC_*50*_ value of quercetin (positive control) was 0.75 ± 0.01 μg/ml (mean ± SE). Multiple comparison was carried out with the method of “Tukey”; and in the same column, means sharing the same letters are not significantly different from each other (*p* > 0.05)*.

The highest antioxidant activity was found for the 50% EtOH extract which imitates medicinal liquor, followed by water extracts. The 95% EtOH extract shows the lowest activity. Our results therefore suggest that traditional goji wine acts as an antioxidant drink, as does goji tea, and do support the recommendation of goji as a natural antioxidant (Donno et al., 2015; Benchennouf et al., 2017).

### Overall Comparison of the Different Regions and Species

**FIGURE 8:**
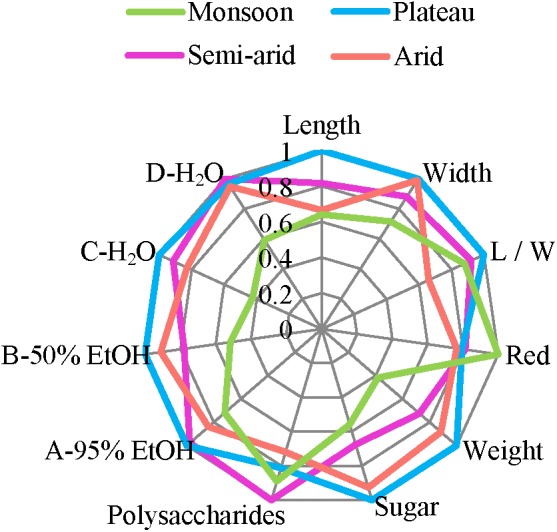
Morphological, chemical, and antioxidant characteristics of goji from different climatic regions. Values are calculated by the ratio relative to the maxims.

**Figure 8** summarizes the above morphological, chemical, and antioxidant data and provides a visual overview of similarities and differences. Overall, *L. chinense* from the monsoon region (blue) differs most while the other regions are more similar, differing among few sensory characteristics only, such as length, weight and sugar contents.

## Conclusion

Ningxia, with a semi-arid climate, always had the reputation of producing best goji quality (a so-called *daodi* production area). But today, new cultivation areas in different climatic regions are recognized as sources of good goji quality too. Depending on the criteria we look at, we find different patterns for goji quality in relation to area of production: Since the Tang Dynasty, goji from semi-arid regions was recognized for its superior quality; however, based on morphological and metabolomic characteristics as well as antioxidative activity it is not possible to separate semi-arid goji from samples from other regions.

Our results do not justify superiority of a specific production area over other areas. Rather, they suggest using goji from different regions for different purposes, based on the specific morphological and chemical traits. For example, large fruits from the plateau are suitable to be marketed as fresh fruits; goji with high sugar content is useful for conserved food; and high antioxidative activity combined with bitterness may suggest medicinal use.

The metabolomic approach combined with morphological analysis and bioactivity evaluation allows for capturing these different goji quality clusters, but, at the same time allows detecting outliers such as different species. This would not be possible with exclusive chemical analysis.

Fruits of *L. barbarum* and *L. chinense* differ in their metabolite profiles including HPTLC flavonoid fingerprint and ^1^H NMR based chemometric analysis, as well as their antioxidant activity. As these two species are used interchangeably as medicinal goji, we suggest treating them as two separate botanical drugs. In case of food use, high sugar content and large fruit size are important, which supports the wider use of *L. barbarum* for food.

Future research on the bioactivity of the two species as well as samples from different cultivation areas beyond China will provide necessary data for specific and most appropriate uses.

## Author Contributions

RY, CW, MH, and YZ developed the concept for the study. RY drafted the paper. CW and MH supervised the study. Plant materials were collected by RY and YC. Morphological traits were measured by XZ and RY. HPTLC was supervised by ER and analyzed by RY. ^1^H NMR was analyzed by RY at MH’s lab and under his supervision. Contents of sugar was measured by RY and YC. Antioxidant activity was measured by YZ and YC. Data were analyzed by RY, YZ, ER, XZ, and YC. All authors revised the paper.

## Conflict of Interest Statement

The authors declare that the research was conducted in the absence of any commercial or financial relationships that could be construed as a potential conflict of interest.

## Footnotes

1 http://www.baidu.com

## Abbreviations

ChP: Chinese Pharmacopoeia
EP: European Pharmacopoeia
HPTLC: High Performance Thin Layer Chromatography
NMR: nuclear magnetic resonance
PCA: principal component analysis
SE: standard error.

## References

[B1] Benchennouf A. Grigorakis S. Loupassaki S. Kokkalou E. (2017). Phytochemical analysis and antioxidant activity of *Lycium barbarum* (Goji) cultivated in Greece. *Pharm. Biol.* 55 596–602. 10.1080/13880209.2016.1265987 27937034 PMC6130502

[B2] Booker A. Frommenwiler D. Johnston D. Umealajekwu C. Reich E. Heinrich M. (2014). Chemical variability along the value chains of turmeric (*Curcuma longa*): a comparison of nuclear magnetic resonance spectroscopy and high performance thin layer chromatography. *J. Ethnopharmacol.* 152 292–301. 10.1016/j.jep.2013.12.042 24417868

[B3] Booker A. Zhai L. Gkouva C. Li S. Heinrich M. (2016). From traditional resource to global commodities:-a comparison of *Rhodiola* species using NMR spectroscopy-metabolomics and HPTLC. *Front. Pharmacol.* 7:254. 10.3389/fphar.2016.00254 27621703 PMC5002433

[B4] Cao Y. L. Wu P. J. (eds) (2015). *Wolfberry Germplasm Resources in China.* Beijing: China Forestry Publishing House.

[B5] Chang R. C. C. So K. F. (2015). *Lycium Barbarum and Human Health.* Dordrecht: Springer.

[B6] Chinese Pharmacopoeia Commission (1963). *Chinese Pharmacopoeia*, Vol. 1 Beijing: The Commercial Press.

[B7] Chinese Pharmacopoeia Commission (2010). *Chinese Pharmacopoeia (English version).* Beijing: China Medical Science Press.

[B8] Chinese Pharmacopoeia Commission (2015a). *Chinese Pharmacopoeia*, Vol. 1 Beijing: China Medical Science Press.

[B9] Chinese Pharmacopoeia Commission (2015b). *Chinese Pharmacopoeia*, Vol. 4 Beijing: China Medical Science Press.

[B10] Dillard C. J. German J. B. (2000). Phytochemicals: nutraceuticals and human health. *J. Sci. Food Agric.* 80 1744–1756.

[B11] Donno D. Beccaro G. L. Mellano M. G. Cerutti A. K. Bounous G. (2015). Goji berry fruit (*Lycium* spp.): antioxidant compound fingerprint and bioactivity evaluation. *J. Funct. Foods* 18 1070–1085. 10.1016/j.jff.2014.05.020

[B12] Donno D. Beccaro G. L. Mellano M. G. Cerutti A. K. Canterino S. Bounous G. (2012). Effect of agronomic and environmental conditions on chemical composition of tree-species buds used for herbal preparations. *Vegetos* 25 21–29.

[B13] Donno D. Boggia R. Zunin P. Cerutti A. K. Guido M. Mellano M. G. (2016a). Phytochemical fingerprint and chemometrics for natural food preparation pattern recognition: an innovative technique in food supplement quality control. *J. Food Sci. Technol.* 53 1071–1083. 10.1007/s13197-015-2115-2116 27162387 PMC4837732

[B14] Donno D. Mellano M. G. Raimondo E. Cerutti A. K. Prgomet Z. Beccaro G. L. (2016b). Influence of applied drying methods on phytochemical composition in fresh and dried goji fruits by HPLC fingerprint. *Eur. Food Res. Technol.* 242 1961–1974. 10.1007/s00217-016-2695-z

[B15] European Directorate for the Quality of Medicines and Healthcare (2017). *European Pharmacopoeia 9. 0.* Strasbourg: Council of Europe, 1263–1264.

[B16] Guo Q. S. (2004). *Cultivation of Medicinal Plants.* Beijing: Higher Education Press.

[B17] Guo S. Duan J. A. Li Y. Wang R. Yan H. Qian D. (2017). Comparison of the bioactive components in two seeds of Ziziphus species by different analytical approaches combined with chemometrics. *Front. Pharmacol.* 8:609. 10.3389/fphar.2017.00609 28928663 PMC5591821

[B18] Hilton J. (2016). “Growth patterns and emerging opportunities in nutraceutical and functional food categories: market overview,” in *Developing New Functional Food and Nutraceutical Products*, eds Bagchi D. Nair S. (San Diego, CA: Academic Press), 1–28.

[B19] Hothorn T. Bretz F. Westfall P. (2008). Simultaneous inference in general parametric models. *Biom. J.* 50 346–363. 10.1002/bimj.200810425 18481363

[B20] Japanese Pharmacopoeia Editorial Committee (2016). *Japanese Pharmacopoeia*, 17th Edn Tokyo: Ministry of Health Labour and Welfare, 1909–1910.

[B21] Knöss W. Chinou I. (2012). Regulation of medicinal plants for public health - European Community monographs on herbal substances. *Planta Med.* 78 1311–1316. 10.1055/s-0031-1298578 22618374

[B22] Korea Food and Drug Administration (2014). *Korean Pharmacopoeia*, 11th Edn, Seoul: Shinil Publishing Company, 1785 1862–1863.

[B23] Lachance P. A. Das Y. T. (2007). “Nutraceuticals,” in *Comprehensive Medicinal Chemistry II*, eds Taylor J. B. Triggle D. J. (Oxford: Elsevier), 449–461.

[B24] Lei J. Liu D. Guo J. (2013). Quality differences of *Lycium barbarum* L. dried fruit in different producing areas. *Xiandai Shipin Keji* 29 494–497.

[B25] Li S. Z. (2003). *Compendium of Materia Medica*, Vol. 5 Beijing: Foreign Language Press, 3185–3194.

[B26] Li X. Lin J. Gao Y. Han W. Chen D. (2012). Antioxidant activity and mechanism of *Rhizoma Cimicifugae*. *Chem. Cent. J.* 6 140. 10.1186/1752-153X-6-140 23173949 PMC3557226

[B27] Liang D. W. (2015). *A Package for Echarts Maps in R.* Available at: http://langdawei.com/REmap/

[B28] Lin L. (2013). *A Comparative Study on the Quality of Lycium barbarum Fruit from Different Producing Areas and Screening of Fine Strains.* Ph.D. thesis, Gansu Agricultural University, Gansu.

[B29] Liu X. X. Liu J. L. Wu N. Xiang Z. J. Liu G. H. Kang J. H. (2015). Study on the contents of secondary metabolites and primary metabolites in different regions of Chinese wolfberry. *Beifang Yuanyi* 23 163–169. 10.11937/bfyy.201523046

[B30] Liu W. Xu J. Zhu R. Zhu Y. Zhao Y. Chen P. (2015). Fingerprinting profile of polysaccharides from *Lycium barbarum* using multiplex approaches and chemometrics. *Int. J. Biol. Macromol.* 78 230–237. 10.1016/j.ijbiomac.2015.03.062 25847838

[B31] Liu Z. Wang D. Li D. Zhang S. (2017). Quality evaluation of *Juniperus rigida* sieb. et zucc. based on phenolic profiles, bioactivity, and hplc fingerprint combined with chemometrics. *Front. Pharmacol.* 8:198. 10.3389/fphar.2017.00198 28469573 PMC5395569

[B32] Ma W. Ni Z. Li H. Chen M. (2008). Changes of the main carotenoid pigment contents during the drying processes of the different harvest stage fruits of *Lycium barbarum* L. *Agric. Sci. Chin.* 7 363–369. 10.1016/S1671-2927(08)60077-60072

[B33] Martinez-Frances V. Hahn E. Rios S. Rivera D. Reich E. Vila R. (2017). Ethnopharmacological and chemical characterization of *Salvia* species used in valencian traditional herbal preparations. *Front. Pharmacol.* 8:467. 10.3389/fphar.2017.00467 28790914 PMC5524814

[B34] Qi G. Su X. Zheng G. Yang J. Bao H. Wang J. (2016). Effect of meteorological factor on fruit growth and accumulation of polysaccharides in *Lycium barbarum*. *Chin. Bull. Bot.* 51 311–321.

[B35] Qian D. Zhao Y. Yang G. Huang L. (2017). Systematic review of chemical constituents in the genus *Lycium* (Solanaceae). *Molecules* 22:E911. 10.3390/molecules22060911 28629116 PMC6152755

[B36] R Core Team (2017). *R: A Language and Environment for Statistical Computing.* Vienna: R Foundation for Statistical Computing.

[B37] Shang Z. J. (2008). *Classic of Shennong’s Herbal with Annotation.* Beijing: Xueyuan Press.

[B38] Shen T. Zou X. Shi J. Li Z. Huang X. Xu Y. (2016). Determination geographical origin and flavonoids content of goji berry using near-infrared spectroscopy and chemometrics. *Food Anal. Methods* 9 68–79. 10.1007/s12161-015-0175-x

[B39] State Forestry Administration of P.R. China (2017). *Homes of Chinese Economic Forest: Forest Medicinal Materials (2017-09-19).* Available at: www.forestry.gov.cn/portal/jlxh/s/3495/content-550359.html

[B40] Su J. (1981). *Xinxiu Bencao.* Hefei: Anhui Science and Technology Press.

[B41] Su Z. S. (2002). History of Zhongning Goji. *Ningxia Hist.* 3 36–40.

[B42] Sun S. M. (1997). “Herbal cultivation and processing,” in *Qianjin Yifang*, 6th Edn, ed. Lu Z. L. (Shenyang: Liaoning Science and Technolgy Press), 138.

[B43] Tang S. W. (1982). *Zhenglei Bencao.* Beijing: People’s Health Press.

[B44] Tao H. J. (1986). *Mingyi Bielu.* Beijing: People’s Health Press.

[B45] Venables W. N. Ripley B. D. (2002). *Modern Applied Statistics with S*, 4th Edn. New York, NY: Springer.

[B46] Walch S. G. Tinzoh L. N. Zimmermann B. F. Stuhlinger W. Lachenmeier D. W. (2011). Antioxidant capacity and polyphenolic composition as quality indicators for aqueous infusions of *Salvia officinalis* L. (sage tea). *Front. Pharmacol.* 2:79. 10.3389/fphar.2011.00079 22194722 PMC3242359

[B47] Wickham H. (2009). *ggplot2: Elegant Graphics for Data Analysis.* New York, NY: Springer-Verlag.

[B48] Wu D. T. Cheong K. L. Deng Y. Lin P. C. Wei F. Lv X. J. (2015). Characterization and comparison of polysaccharides from *Lycium barbarum* in China using saccharide mapping based on PACE and HPTLC. *Carbohydr. Polym.* 134 12–19. 10.1016/j.carbpol.2015.07.052 26428094

[B49] Wu Q. J. (1959). *Zhiwu Mingshi Tukao Changbian.* Beijing: Commercial Press, 1063–1067.

[B50] Wu Z. Y. Raven H. P. Hong D. Y. (1994). *Flora of China*, Vol. 17 Beijing: Science Press, 301–304.

[B51] Xiao X. H. Chen S. L. Huang L. Q. Xiao P. G. (2009). Survey of investigations on daodi Chinese medicinal materials in China since 1980s. *Zhongguo Zhong Yao Za Zhi* 34 519–523. 19526774

[B52] Xie J. H. Tang W. Jin M. L. Li J. E. Xie M. Y. (2016). Recent advances in bioactive polysaccharides from *Lycium barbarum* L., *Zizyphus jujuba* Mill, *Plantago* spp., and *Morus* spp.: structures and functionalities. *Food Hydrocoll.* 60 148–160. 10.1016/j.foodhyd.2016.03.030

[B53] Xin T. Yao H. Gao H. Zhou X. Ma X. Xu C. (2013). Super food *Lycium barbarum* (Solanaceae) traceability via an internal transcribed spacer 2 barcode. *Food Res. Int.* 54 1699–1704. 10.1016/j.foodres.2013.10.007

[B54] Xu C. Q. Liu S. Xu R. Chen J. Qiao H. L. Jin H. Y. (2014). Investigation of production status in major wolfberry producing areas of China and some suggestions. *Zhongguo Zhong Yao Za Zhi* 39 1979–1984. 10.4268/cjcmm20141106 25272826

[B55] Yao R. Heinrich M. Weckerle C. S. (2018a). The genus *Lycium* as food and medicine: a botanical, ethnobotanical and historical review. *J. Ethnopharmacol.* 211 50–66. 10.1016/j.jep.2017.10.010 29042287

[B56] Yao R. Huang C. Chen X. Yin Z. Fu Y. Li L. (2018b). Two complement fixing pectic polysaccharides from pedicel of *Lycium barbarum* L. promote cellular antioxidant defense. *Int. J. Biol. Macromol.* 112 356–363. 10.1016/j.ijbiomac.2018.01.207 29409772

[B57] Yao X. Peng Y. Xu L. J. Li L. Wu Q. L. Xiao P. G. (2011). Phytochemical and biological studies of *Lycium* medicinal plants. *Chem. Biodivers.* 8 976–1010. 10.1002/cbdv.201000018 21674776

[B58] Yao X. Peng Y. Zhou Q. Xiao P. Sun S. (2010). Distinction of eight Lycium species by Fourier-transform infrared spectroscopy and two-dimensional correlation IR spectroscopy. *J. Mol. Struct.* 974 161–164. 10.1016/j.molstruc.2010.02.064

[B59] Zhang K. Y. Leung H. W. Yeung H. W. Wong R. N. (2001). Differentiation of *Lycium barbarum* from its related *Lycium* species using random amplified polymorphic DNA. *Planta Med.* 67 379–381. 10.1055/s-2001-14310 11458465

[B60] Zhang L. Zheng G. Teng Y. Wang J. (2012). Comparison research on fruit quality of *Lycium barbarum* L. in different regions. *Northwest Pharm. J.* 27 195–197. 10.3969/j.issn.1004-2407.2012.03.003 22512203

[B61] Zhang Q. Chen W. Zhao J. Xi W. (2016). Functional constituents and antioxidant activities of eight Chinese native goji genotypes. *Food Chem.* 200 230–236. 10.1016/j.foodchem.2016.01.046 26830583

[B62] Zhao Z. Guo P. Brand E. (2012). The formation of daodi medicinal materials. *J. Ethnopharmacol.* 140 476–481. 10.1016/j.jep.2012.01.048 22342382

[B63] Zou Y. Chen X. Yang W. Liu S. (2011). Response surface methodology for optimization of the ultrasonic extraction of polysaccharides from *Codonopsis pilosula* Nannf.*var.modesta* L.T.Shen. *Carbohydr. Polym.* 84 503–508. 10.1016/j.carbpol.2010.12.013

[B64] Zou Y. F. Ho G. T. Malterud K. E. Le N. H. Inngjerdingen K. T. Barsett H. (2014). Enzyme inhibition, antioxidant and immunomodulatory activities, and brine shrimp toxicity of extracts from the root bark, stem bark and leaves of *Terminalia macroptera*. *J. Ethnopharmacol.* 155 1219–1226. 10.1016/j.jep.2014.07.004 25017373

